# Supplementary material for: COVID-19 vaccine effectiveness in preventing severe outcomes and assessing the impact of prior SARS-CoV-2 infection among hospitalized adults in Albania, July 2022-July 2023
Source: PLOS Glob Public Health. 2026 May 12;6(5):e0006418. doi: 10.1371/journal.pgph.0006418 (PMC13166910; doi:10.1371/journal.pgph.0006418)
Supplement: Attachment — Submitted filename: Response to reviewers.docx [file pgph.0006418.s002.docx]

Dear Editor and Reviewers,

Thank you for your careful evaluation of our manuscript and for the constructive comments, which have helped improve the clarity, rigor, and relevance of the study. Below we provide a detailed, point-by-point response. All reviewer comments are reproduced in bold, followed by our responses.

**Editor**

We confirm that the underlying data cannot be publicly shared due to legal restrictions, as they originate from official national surveillance information systems held by a governmental public health authority and contain sensitive health information. To support transparency and reproducibility to the extent permitted, we have provided the digitized data points extracted from the figure used in the analyses as Supporting Information.

In addition, we have amended the Data Availability Statement to explicitly describe the legal restrictions on data sharing and to include the contact details of the Institute of Public Health (IPH), Tirana, Albania, as the data-owning authority for researchers who meet the criteria for access to national data.

We have reviewed and corrected the figure files to meet PLOS technical requirements and verified compliance using the PLOS NAAS tool prior to upload. Regarding point 6, we confirm that we would like to opt in to publishing the peer review history of our article.

**Reviewer #1**

**Minor Comments**

**Abstract: The phrase “VE against severe outcomes was 30.6%, increasing to 37.8% with a booster” could be misinterpreted; could the authors clarify if these are adjusted estimates? Starting the sentence in the 'results section' with the full meaning of VE is more acceptable.**

We thank the reviewer for this helpful comment. To avoid any potential misinterpretation, we now explicitly define VE at first mention as adjusted vaccine effectiveness (VE) in the abstract and maintain this terminology consistently throughout the manuscript.

**Introduction: Can the authors consider an Albania-specific background after a short intro of the global COVID-19 background?**

We have added an Albania-specific background section to provide national context for the study. This addition summarizes seroprevalence findings and the rollout of the national COVID-19 vaccination campaign. We also describe vaccination coverage, priority groups, and the vaccines used in Albania.

**References: Can the authors kindly update with the latest WHO SAGE recommendations on hybrid immunity and booster strategies?**

The reference list has been updated to include the latest WHO Strategic Advisory Group of Experts on Immunization (SAGE) recommendations regarding hybrid immunity and booster vaccination strategies. The manuscript text has been revised accordingly to ensure alignment with current WHO guidance.

**Major Comments**

**1. Methods**

**In the description of inclusion/exclusion criteria, Fig 1 is mentioned but not fully described in the text.**

The description of the inclusion and exclusion criteria has been expanded in the Methods section to align with Figure 1. We now describe the eligibility criteria for SARI patients aged ≥18 years with available SARS-CoV-2 RT-PCR results and documented COVID-19 vaccination status. In addition, we detail the number of excluded patients and the specific reasons for exclusion, and report the composition of the final analytic cohort.

**Can the authors kindly clarify how the documented co-morbidities were confirmed (through self-report or medical records, or any other means)?**

The manuscript has been revised to state that co-morbidities were ascertained from hospital medical records at the time of admission. The definition of co-morbidities has been updated in the Methods section.

**2. VE Estimates**

**Some confidence intervals appear wide & with negative values, which presupposes a limited sample size and/or statistical uncertainty, can the authors clarify.**

Thank you for underlining out this point, we acknowledge that several vaccine effectiveness estimates, particularly in subgroup analyses, are accompanied by wide confidence intervals, in some cases crossing the null value. To address this, we have revised the manuscript to emphasize this uncertainty. Specifically, in the Abstract many VE estimates have wide confidence intervals overlapping zero, indicating considerable uncertainty and lack of statistical significance in some subgroup analyses. In the Discussion, is mentioned caution that these findings should be interpreted as observational point estimates rather than definitive evidence, while noting that the direction of the estimates is biologically plausible and consistent with enhanced protection from booster doses. Finally, in the Conclusion, we stated that modest VE estimates within the Albanian setting, highlighting factors such as immune evasion, waning immunity, and population immunity from prior infection.

**There appears to be a relatively low VE compared to other European studies, can the authors comment on possible reasons e.g. due to vaccine types used, coverage rates, Omicron predominance, etc).**

We have expanded the Discussion to contextualize the relatively lower VE estimates observed in our study compared with other European settings. We now note that our study period coincided with Omicron-dominant circulation, during which VE against hospitalization has been consistently lower than in earlier variant periods.

In addition, we highlight several Albania-specific factors that may have contributed to the modest VE estimates, including the use of a heterogeneous vaccine product mix (mRNA, viral vector, and inactivated vaccines), lower vaccination and booster coverage in the hospitalized SARI population, and a high proportion of unvaccinated individuals.

**3. A Priori Infection Analysis**

**The authors describe an increase in VE when combined with prior infection (76.2%). Please could the authors expand on this in the discussion about hybrid immunity and perhaps policy implications, especially in settings with low booster uptake.**

In the discussion we explain this finding within the existing literature and, in addition, we have added a policy-focused paragraph discussing the implications of hybrid immunity in settings with low booster uptake, such as Albania.

**4. Limitations**

**The manuscript acknowledges limited viral typing capacity. Could the authors expand on how this may affect VE estimates, given Omicron sub-variant diversity in the limitations section?**

The Limitations section has been expanded to explain how the lack of comprehensive genomic sequencing may influence our VE estimates. We now clarify that the absence of variant- and sub-variant–specific stratification means that the reported VE likely represents an average effect across multiple Omicron sub-variants circulating during the study period.

**Can the authors describe potential biases from hospital stay duration as a proxy for severity?**

The Limitations section has been updated to address potential biases from using hospital length of stay as a proxy for disease severity. We note that while length of stay was included in adjusted models to partially account for differences in clinical course, it remains an imperfect severity measure, as it may be influenced by non-clinical factors and competing outcomes.

**Reviewer #2**

**Major Issues**

**1. Statistical Precision and Confidence Intervals: A primary concern is the statistical power of the study. Many VE estimates feature very wide confidence intervals (CIs) that cross the null value or include negative values. In clinical and public health research, when a confidence interval for VE crosses zero, the result is not considered statistically significant, meaning the study cannot definitively conclude that the vaccine provided protection in that specific subgroup. The authors should explicitly acknowledge this lack of significance in the discussion sections. Presenting these point estimates as definitive "protective effects" without emphasizing the uncertainty (the wide CIs) is misleading.**

Thank you for this helpful comment. We have revised the Discussion to explicitly acknowledge the limited statistical precision of several subgroup analyses, emphasizing that VE estimates with confidence intervals crossing the null value do not represent statistically significant protection. Language throughout the Discussion was modified to avoid presenting point estimates as definitive effects, and results are now framed as observational estimates requiring cautious interpretation.

**2.Discrepancy in VE Values: The reported VE of 30–38% is considerably lower than the 70–90% often reported in other European studies during similar periods. While the authors mention rollout timing, a deeper analysis is required regarding Vaccine Product Mix and Variant Dynamics.**

We have revised the Discussion and Limitations sections to address these issues. Specifically, we now note that vaccine product–specific VE analyses were not performed, precluding estimation of VE by vaccine platform in a setting with a heterogeneous vaccine mix. In addition, the absence of comprehensive genomic sequencing data prevented stratification of VE by SARS-CoV-2 variants or Omicron sub-variants, which differ in immune escape potential. We clarify that the reported VE estimates therefore represent averages across vaccine products and circulating Omicron sub-variants, which may have attenuated overall effectiveness.

**Minor Issues**

**1.Table clarity and data presentation**

**Table 1 Formatting: The "Age" row currently presents "53-74" and "66" in a single cell, which is highly confusing. This must be reformatted to clearly distinguish between the Median and the Interquartile Range (IQR).**

We have reformatted Table 1 so that age is now reported as median (IQR), with the interquartile range clearly indicated in brackets.

**Inconsistency: The number of decimal points varies throughout the tables. For professional reporting, please standardize to one or two decimal places consistently.**

We have standardized numerical reporting across the tables and corresponding text to one decimal place to ensure consistent presentation throughout the manuscript.

**2.Over-interpretation of results: The authors describe VE estimates of 30–38% as "substantial." In the context of global COVID-19 research, where "substantial" is typically reserved for effectiveness above 60–70%, this characterization is an over-interpretation. A more neutral term would be more scientifically accurate given the data provided.**

We have revised the Discussion and Conclusion to use neutral language throughout. VE estimates in this range are now referred to as modest point estimates, and their interpretation is consistently qualified by wide confidence intervals and the observational nature of the study.

**Global Public Health Framing: To increase the manuscript's impact, the authors should broaden the discussion beyond the Albanian context.**

We have revised the Discussion and Conclusion to explicitly situate our findings within a broader global public health context. Beyond the Albanian setting, we now frame the results as relevant to mid-income countries with heterogeneous vaccine product mixes, moderate primary-series uptake, limited booster coverage, and Omicron-era transmission.

We again thank the Editor and Reviewers for their thoughtful comments and believe that the revisions have strengthened the manuscript substantially.

Sincerely on behalf of all co-authors,

Jonilda Sulo
