# Supplementary material for: COVID-19 vaccine effectiveness in preventing severe outcomes and assessing the impact of prior SARS-CoV-2 infection among hospitalized adults in Albania, July 2022-July 2023
Source: PLOS Glob Public Health. 2026 May 12;6(5):e0006418. doi: 10.1371/journal.pgph.0006418 (PMC13166910; doi:10.1371/journal.pgph.0006418)
Supplement: Attachment — Submitted filename: Response__reviewers_rev2.docx [file pgph.0006418.s003.docx]

**Dear Editor,**

Thank you for the opportunity to revise our manuscript, we appreciate the careful assessment and the constructive comments provided by you and the reviewers. We have revised the manuscript accordingly and believe the changes have substantially improved its clarity, methodological transparency, and interpretation.

In response to the comments received, we have made the following key revisions:

**MAJOR COMENTS**

**1. Exclusion of influenza-positive cases (line 115). You mention excluding influenza-positive cases from the analysis. Could you please provide additional justification for this decision?**

**A citation supporting this analytic approach, or**

**A sensitivity analysis in which influenza-positive cases are included (e.g., as negative controls) to assess how this impacts your primary results.**

**Given that many of your estimates are non-significant, I would not expect large shifts, but including such an analysis would strengthen the methodological rigor and reassure readers that findings are robust to this specification.**

We followed the suggestion to add a supporting methodological reference relevant to the exclusion of influenza-positive cases from the primary analysis: Doll et al., *Effects of Confounding Bias in Coronavirus Disease 2019 (COVID-19) and Influenza Vaccine Effectiveness Test-Negative Designs Due to Correlated Influenza and COVID-19 Vaccination Behaviors*. This reference has been incorporated into the revised manuscript.

**2. Contextualization of COVID-19 burden in Albania (lines 41 and related discussion). I appreciate that you have added additional information about the epidemic in Albania, as requested by prior reviewers. However, I think further contextualization would strengthen the manuscript.**

We have further expanded the Introduction to better contextualize the COVID-19 burden in Albania. Specifically, we now indicate that 319959 confirmed cases correspond to approximately 11.4% of the Albanian population. We also more explicitly distinguish confirmed case counts from likely cumulative infection burden by contextualizing the already cited Albanian seroprevalence evidence showing that more than 90% of the population had developed SARS-CoV-2 antibodies by mid-2022. In addition, we clarify that with continued Omicron transmission, the proportion of the population with prior infection or hybrid immunity during 2022–2023 was likely higher than indicated by confirmed case counts alone.

**3. Timing of comparator studies (Discussion section). When comparing your vaccine effectiveness estimates to other studies, could you please specify the dates during which those studies were conducted (not just publication dates)? Vaccine effectiveness varies substantially by circulating variant and calendar time, so indicating when comparator studies took place would help readers better contextualize similarities and differences.**

We have revised the Discussion to specify the study periods of the comparator studies cited, rather than relying only on publication dates.

**4. Clarification of “variants not included” (line 90). In the Methods, you state that variants were “not included.” Could you clarify what this means operationally?**

We have revised the Methods section to state explicitly that variant-specific data were not available at the individual case level because genomic typing was not performed systematically for all SARS-CoV-2–positive SARI patients during the study period. We further clarify that all laboratory-confirmed SARS-CoV-2 cases were therefore analysed together as COVID-19–positive cases without stratification by variant or sub-variant.

**5. Analysis of severe outcomes. For your vaccine effectiveness analysis of severe outcomes, could you provide more detail on how these models were structured? How were non-severe cases handled in that analysis (excluded, treated as controls?)?**

We have expanded the Statistical Analysis section to clarify how subgroup models, including severe outcome analyses, were structured. Specifically, we now state that each subgroup analysis was conducted within a dedicated cohort restricted to the population of interest, and that within each subgroup-specific cohort the test-negative design was applied, with SARS-CoV-2–positive SARI patients classified as cases and SARS-CoV-2–negative SARI patients as controls.

**MINOR COMMENTS**

**1. Use of causal language (Abstract conclusion and elsewhere). In the abstract and elsewhere, you use the word “effect.” Because this is an observational study, I recommend avoiding causal language. Unless referring specifically to “vaccine effectiveness” as a standard epidemiologic term, I suggest replacing “effect” with “association” or similar non-causal terminology throughout the manuscript.**

We have revised the manuscript to avoid causal language where inappropriate for an observational study, except where “vaccine effectiveness” is used as a standard epidemiologic term. Terms such as “effect” and “protective effect” were replaced with more neutral, non-causal wording where appropriate.

**2. Clarification of Comirnaty (lines 50–57) - clarify if this is a brand name, manufacturer, or product designation.**

We have clarified this in the manuscript by specifying Comirnaty (BNT162b2, Pfizer-BioNTech) at first mention.

**3. Table 1 – Mean vs. median. In Table 1, you list a mean but later describe median and interquartile range (IQR) in your response to reviewers.**

We have clarified this by adding the acronym IQR at the first mention of interquartile range in the text, and Table 1 presents median (IQR) consistently.

**4. Table 2 – Confidence interval formatting. In Table 2 (third row), part of a confidence interval appears to be missing.**

We corrected the incomplete confidence interval in Table 2. This error resulted from a spreadsheet formula/formatting issue, and the full confidence interval is now presented correctly.

**5. Language comparing vaccine effectiveness estimates (line 214 and following paragraph)
When comparing vaccine effectiveness estimates (e.g., primary course 30.1% vs. booster 31.4%), I suggest avoiding language such as “higher” or “lower” when the estimates are numerically similar and overlapping. Simply presenting the estimates side-by-side without comparative wording would be clearer and more neutral. The same applies to the following paragraph.**

We have revised the relevant sentences in the Results and Discussion to avoid comparative wording such as “higher,” “lower,” and “increasing” where vaccine effectiveness estimates were numerically similar and had overlapping confidence intervals. These estimates are now presented side-by-side in a more neutral and descriptive manner.

**6. Formatting of large numbers. Please ensure consistency in formatting numbers with four or more digits (e.g., inclusion or exclusion of commas in the thousands place). Choose one format and apply it consistently throughout.**

We have reviewed the manuscript and standardized the formatting of all numbers with four or more digits by consistently presenting them without commas throughout the text and tables, in line with the formatting used in the figures.

We hope that these revisions satisfactorily address the remaining concerns and improve the manuscript. We thank you again for your careful consideration.

**Sincerely,**

Jonilda Sulo

on behalf of all authors
